# Host Specialisation, Immune Cross-Reaction and the Composition of Communities of Co-circulating Borrelia Strains

**DOI:** 10.1007/s11538-021-00896-2

**Published:** 2021-05-03

**Authors:** Ben Adams, Katharine S Walter, Maria A. Diuk-Wasser

**Affiliations:** 1 Department of Mathematical Sciences, University of Bath, Bath, UK; 2 Division of Infectious Disease and Geographic Medicine, Stanford School of Medicine, Stanford, USA; 3 Department of Ecology, Evolution and Environmental Biology, Columbia University, New York, USA

**Keywords:** Borrelia, Strain, Diversity, Niche, Specialist, Generalist, Cross-immunity

## Abstract

We use mathematical modelling to examine how microbial strain communities are structured by the host specialisation traits and antigenic relationships of their members. The model is quite general and broadly applicable, but we focus on *Borrelia burgdorferi*, the Lyme disease bacterium, transmitted by ticks to mice and birds. In this system, host specialisation driven by the evasion of innate immunity has been linked to multiple niche polymorphism, while antigenic differentiation driven by the evasion of adaptive immunity has been linked to negative frequency dependence. Our model is composed of two host species, one vector, and multiple co-circulating pathogen strains that vary in their host specificity and their antigenic distances from one another. We explore the conditions required to maintain pathogen diversity. We show that the combination of host specificity and antigenic differentiation creates an intricate niche structure. Unequivocal rules that relate the stability of a strain community directly to the trait composition of its members are elusive. However, broad patterns are evident. When antigenic differentiation is weak, stable communities are typically composed entirely of generalists that can exploit either host species equally well. As antigenic differentiation increases, more diverse stable communities emerge, typically around trait compositions of generalists, generalists and very similar specialists, and specialists roughly balanced between the two host species.

**Supplementary Information:**

The online version contains supplementary material available at 10.1007/s11538-021-00896-2.

## Introduction

Pathogens often exhibit substantial variation in virulence, transmissibility, susceptibility to drugs and other traits. What maintains pathogen diversity—and how public health interventions may shape standing pathogen diversity—is a critical epidemiological question (Greischar et al. 2020). Natural populations of *Borrelia burgdorferi*, the human Lyme disease bacterium, are characterised by high levels of co-circulating antigenic diversity. The mechanisms that shape and maintain this diversity are not well understood. Here, we develop an eco-epidemiological model to explore how host specificity and antigenic differentiation may characterise the co-circulating communities of strains within a Borrelia species.

*Borrelia burgdorferi* (Bb) sensu lato is a complex of bacterial species found throughout Europe and North America. More than 20 different genospecies of the complex have been identified so far. The epidemiological dynamics are zoonotic, but six species are known to cause Lyme disease in humans: *B. burgdorferi sensu stricto (s.s.), B. afzelii, B. garinii, B. spielmanii, B. bavariensis* (formerly referred to as *B. garinii* OspA serotype 4) and *B. mayonii* (Lin et al. 2020). Bb spirochotes are transmitted between vertebrate hosts by *Ixodes* spp. ticks. Important transmission-competent hosts include small rodents and birds. In many regions, there is a diverse community of co-circulating Bb strains and/or species. It has been suggested that key mechanisms governing the composition of these communities are multiple niche polymorphism (MNP) and negative frequency dependence (NFD) (Kurtenbach et al. 2002, 2006). MNP can occur when Borrelia strains are specialised to exploit particular vertebrate host species, mostly likely by producing proteins to evade host complement (Lin et al. 2020). Then, different host species form distinct ecological niches. NFD can occur when infection with one Bb strain elicits an adaptive immune response in the host that provides at least partial protection against re-infection with that strain and secondary infection with other antigenically similar strains. Then, as the prevalence (or frequency) of a strain increases, the size of the host population that can be infected with similar strains decreases. There is a good body of empirical and theoretical work on how MNP and NFD may separately determine microbial community compositions. Here, we develop this theory using a mathematical model to examine how Bb communities may be structured by the combined effects of host specialization and immune cross-protection, given that transmission occurs via a generalist vector. We begin with a more detailed review of the multiple niche polymorphism and negative frequency dependence hypotheses.

### Multiple Niche Polymorphism

The hypothesis that multiple niche polymorphism shapes Bb strain communities is supported by empirical evidence that strains have different host specialisation traits. This characteristic is often related to the Bb ospC surface proteins which are polymorphic and associated with the evasion of innate host immunity (Grimm et al. 2004; Tilly et al. 2006; Carrasco et al. 2015; Hartmann et al. 2006). In Europe, some Bb species are known to be specialised to particular mammalian or avian host species (Hanincová et al. 2003b, a). In North America, field studies have found positive associations between different Bb strains and host species (Brisson and Dykhuizen 2004; Brisson et al. 2008; Vuong et al. 2014; Hanincová et al. 2006; Mechai et al. 2016; Vuong et al. 2017; Brinkerhoff et al. 2010). Laboratory studies have found differential fitness of Bb strains in different host species (Hanincová et al. 2008; Derdáková et al. 2004; Mather et al. 1989; Ginsberg et al. 2005). We may think of co-circulating Bb strains as a community exploiting a resource landscape composed of various host species. Generalist and specialist strains access different resource niches, and multiple niche polymorphism occurs when these strains coexist.

General theory for the coexistence of generalists and specialists in ecological communities is well developed. Most of this theory is informed by mathematical modelling and focuses on conditions for the coexistence of a generalist and two specialists in an environment composed of two resource types. In broad terms, specialists exploit a narrow range of resource types very efficiently, while generalists exploit a wide range of resource types less efficiently. Criteria for coexistence tend to depend on the details of model structure. Wilson and Yoshimura (1994) show that specialists are only viable if generalism incurs a disproportionately high cost. In their framework, the stable coexistence of a generalist and two specialists additionally requires flexible resource preference and temporal variation in resource abundance. Hochberg and Holt (1990) focus on specialism in parasites. In their framework, coexistence of any two strains requires that one is more transmissible (i.e. a better disperser), while the other is dominant within multiply infected hosts. They also find that within-host interference or facilitation can mean that the order in which parasite strains are introduced into a host population determines the final community composition. The work of Wilson and Yoshimura (1994) is revisited in several studies. Egas et al. (2004) show that even if a community formed of a generalist and two specialists is ecologically stable, it may not be evolutionarily stable. Abrams (2006) show that coexistence can be achieved with less restrictive conditions on temporal variation and resource abundance if there is flexible resource preference. Then, Nagelkerke and Menken (2013) show that coexistence can be achieved without any temporal variation or flexible resource preference if there is spatial variation in the environment and the generalist can exploit more habitat types than the specialists.

In summary, theory suggests that specialists should exclude generalists in time-constant, homogeneous environments because they are, by assumption, fitter. However, generalists may coexist with, or exclude, specialists if they have flexible resource preference and the environment varies with time, or if the environment varies in space. For Borrelia, the environment is composed of the tick and host populations. Since the tick vectors are generalists, there seems little scope for flexible resource preference. In fact, the presence of a generalist vector introduces a clear fitness cost for specialist Borrelia strains when ticks feed on the non-preferred host species. It is, however, highly likely that the host and tick populations are spatially structured. They are also subject to several strong seasonal drivers with intricate phase relationships that generate a complex pattern of environmental time variation that may also have a role in shaping strain communities. In this paper, however, we will maintain a sharp focus on how an additional niche dimension, in terms of antigenic variation, affects the coexistence of specialists and generalists by considering constant and homogeneous host populations.

### Negative Frequency Dependence

The hypothesis that negative frequency dependence shapes Bb strain communities is supported by empirical evidence for a cross-protective vertebrate antibody response. This is often related to the Bb ospC surface protein. OspC alleles fall into discrete clusters, termed ospC major groups (OMGs). The genetic divergence within each cluster is less than 2%; between clusters it is at least 8% (Wang et al. 1999). It has been shown that cross-protection between strains in the same OMG is strong, but cross-protection between strains in different OMGs is weak or absent (Gilmore et al. 1996; Probert et al. 1994, 1997; Earnhart et al. 2005). Hence, acquired host immunity induces indirect competition between Bb strains that may influence community composition (Wang et al 1999; Lagal et al. 2003; Durand et al. 2015). As before, if we think of co-circulating Bb strains as a community exploiting a resource landscape composed of host species, then each strain degrades the resource available to other strains in the same antigenic cluster. Consequently, there is temporary selection against high prevalence strains which, in some circumstances, could lead to cyclic variation of dominant groups.

Theory for the coexistence of pathogen strains under cross-protective immunity is well developed. Much of this work has focused on understanding how the influenza virus evolves to evade acquired immunity. But studies have also looked at the strain dynamics for dengue, neisseria and malaria, and in more general contexts. An in depth review of the main modelling approaches can be found in Kucharski et al. (2016). Here, we highlight some insightful results regarding strain coexistence. Outcomes tend to depend on the detailed model assumptions, for instance regarding age structure and spatial structure. Furthermore, strong artificial structures such as symmetry assumptions are frequently imposed to gain mathematical tractability. Castillo-Chavez et al. (1989) focused on two strains. In their framework, stable coexistence occurs under partial cross-immunity, but age structure has a destabilising effect that induces oscillatory coexistence. Andreasen et al. (1997) considered more than two strains. In their model, communities of three strains generally coexist, but communities of four or five strains are either unstable or oscillatory. Subsequent models by Ferguson and Andreasen (2002) and Gog and Swinton (2002) also found stable communities of four strains with bistability of different community structures and, in some frameworks, coexistence with oscillatory or chaotic dynamics. Adams and Sasaki (2007) examined the role of the function used to model the relationship between antigenic similarity and the strength of cross-immunity. They show that community stability depends on the convexity of this function.

In summary, theory suggests that the complex feedbacks of negative frequency dependence lead to an intricate and fluid landscape of antigenic niches. Within the same model framework, it may be possible to fill the entire antigenic space with a small number of strains in broad niches, or a large number of strains in narrow niches. For some models, this picture may be further complicated by bistability and oscillatory dynamics. For Borrelia, the nature of the antigenic space and the mapping between antigenic similarity and cross-immunity are not known. Nevertheless, observations such as the ospC clustering provide strong empirical evidence that antigenic niches are important. In this paper, therefore, we will use a relatively simple model for antigenic structure in order to maintain transparent mechanisms.

Here, we explore the hypothesis that both MNP and NFD are operating in the Borrelia system. So the trait space of host specialisation to evade innate immunity is extended into an additional dimension by antigenic differentiation to evade adaptive immunity. We explore this hypothesis using a mathematical model that integrates host specialisation and immune cross-protection into a relatively simple eco-epidemiological framework for Borrelia transmission. We give a detailed account of the model in the next section.

## Mathematical Model

### Eco-epidemiological Processes

Borrelia eco-epidemiology typically involves transmission between vertebrate hosts via the bites of *Ixodes* tick larvae and nymphs. The life cycle of *Ixodes* ticks has egg, larva, nymph and adult stages (Anderson and Magnarelli 2008; Barbour and Fish 1993). Progression from larva to nymph and nymph to adult, and the production of eggs by adults, requires host blood meals. Usually, the tick remains attached to a single host for several days and then drops off to moult to the next stage. The moult may be completed within a few weeks, or delayed for several months over winter. Larvae are generally uninfected with Bb when they hatch (Patrican 1997). They may acquire infection when taking a blood meal from an infected host and transmit this to another host as a nymph. Bb infection persists into the adult stage. But adults mainly feed on white-tailed deer (Wilson et al. 1985) which are non-competent for Bb transmission (Telford et al. 1988). More generally, diverse vertebrate communities, which include both competent and non-competent hosts, may lead to a ‘dilution effect’ where Bb transmission is ‘diluted’ by the presence of non-competent hosts which serve as transmission dead ends (LoGiudice et al. 2003).

### Existing Models for Tick-Borne Zoonoses

There are numerous models for tick-borne zoonoses in the literature. Most include one or more vertebrate host species and a tick population partitioned into several life stages. This basic structure is refined or extended in various ways depending on the focus of the study.

Porco (1999) examines the temporal overlap of tick and host cohorts and the relative timing of control events. The model is discrete time with a projection interval of one month. Ticks in all life stages are additionally classified according to when they last fed. The study shows that tick control in spring is most effective for interrupting transmission. Norman et al. (1999) examine the effect of dilution on epidemic risk, characterised by the basic reproduction number. The model is continuous time. The study shows that moderate densities of non-competent hosts increase the epidemic risk by amplifying the tick population, but high densities of non-competent hosts reduce the epidemic risk by transmission dilution. Rosà et al. (2003) and Hartemink et al. (2008) examine the contribution of different transmission pathways to the epidemic risk. Rosà et al. (2003) use a version of the Norman et al. (1999) model, and Hartemink et al. (2008) focus on a process-based construction of the basic reproduction number. Both models include co-feeding transmission between ticks based on assumed distributions for tick aggregation on hosts. The study by Hartemink et al. (2008) suggests that *Borrelia burgdorferi* is primarily sustained by systemic transmission from the host; direct transmission between co-feeding ticks is of less importance. Haven et al. (2012) examine the trade-off between early infectivity and infection persistence. The model is mixed discrete and continuous time. The study shows that rapidly cleared strains dominate persistent strains if tick larva and nymphs quest synchronously. Ogden et al. (2013) examine the geographic spread of infection. The model is continuous time with the host and tick populations subject to seasonal drivers. The study shows the spread of ticks to a region is likely to precede the appearance of Borrelia by around five years. Lou and Wu (2017) offer a thorough and insightful review of recent mathematical modelling studies that have examined many important aspects of Borrelia eco-epidemiology including tick stage structure, dilution and amplification when there are several host species, seasonality and co-infection with other bacterial pathogens. Nguyen et al. (2019) examine how several ecological factors affect the presence and prevalence of Borrelia. They use a continuous-time model that incorporates mouse and deer hosts, tick life history and host preference and seasonal variation in elements such as tick biting behaviour, tick mortality and deer reproduction. The study shows that tick host preference is unlikely to be a significant factor in Borrelia epidemiology, infection prevalence is positively correlated with the duration of the tick biting season, and deer are ineffective at dispersing Borrelia to other regions because they have a minor role in transmission.

### Model Description

Our model is based on the continuous-time framework proposed by Norman et al. (1999) and studied by Rosà et al. (2003). We modify this framework to include two host species and multiple co-circulating pathogen strains.

#### Mouse and Bird Demography

We model the mouse population *M* as homogeneous and well mixed. Mice are born at rate $\mu _M \bar{M}$ and die at per capita rate $\mu _M$. So the size of the population remains constant at $\bar{M}$. This is a simpler assumption than in Norman et al. (1999), where the birth rate is density dependent according to a logistic model. We model the bird population *B* in the same way, with parameters $\mu _B$ and $\bar{B}$.

#### Tick Demography

We partition the tick population to reflect basic life history into larvae *L*, and nymphs *N*. We assume that the tick birth rate is independent of the adult population size, and adults have no role in infection transmission. Therefore, we do not model the adult tick population. Tick larvae hatch at constant rate $\mu _T\bar{T}$, and ticks in all life stages die at rate $\mu _T$. Mice and birds encounter larvae or nymphs at density-dependent rates $\lambda _M (L + N)$ and $\lambda _B (L + N)$. Proportions $L/(L + N)$ and $N/(L + N)$ of these encounters are with, respectively, larvae and nymphs. An encounter between a tick and a host results in the tick progressing to the next life history stage, larva to nymph, nymph to adult. A proportion $\delta$ of larvae successfully complete this transition. The remainder die during the moult. For simplicity, we assume that the transition occurs instantaneously. Parameter values are given in Table 1. These are reasonable order of magnitude estimates for the Borrelia system. The objective of this study is to gain qualitative insight, so precise parameter values are not required.

**Table 1:** Parameter descriptions and values

Parameter	Description	Value
$\mu _M$	Mouse per capita birth/death rate	1
$\bar{M}$	Mouse reproductive population size	500
$\mu _B$	Bird per capita birth/death rate	1
$\bar{B}$	Bird reproductive population size	500
$\mu _T$	Tick per capita birth/death rate	1
$\bar{T}$	Tick reproductive population size	50,000
$\lambda _M$	Encounter rate per mouse, per tick	0.0012
$\lambda _B$	Encounter rate per bird, per tick	0.0012
$\delta$	Probability of successful moult	0.9
$\xi$	Baseline/overall host–tick transmission probability	0.7
$\sigma$	Location in antigenic space	0–2
$\omega$	Host specialisation	0–1

All rates are per year

#### Borrelia Strains

We model multiple co-circulating Borrelia strains. Each strain is characterised by two traits. Host specialisation is parameterised by $\omega$ where $0 \le \omega \le 1$. A strain with $\omega = 0$ can only infect birds. A strain with $\omega = 1$ can only infect mice. A strain with $\omega = 0.5$ is equally able to infect mice and birds. We refer to strains with $0< \omega < 0.33$ as strong bird specialists, strains with $0.67< \omega < 1$ as strong mouse specialists and other strains as weak specialists or generalists. The intervals in these definitions were determined empirically, as discussed in Sect. 4.2.2.

**Fig. 1:**
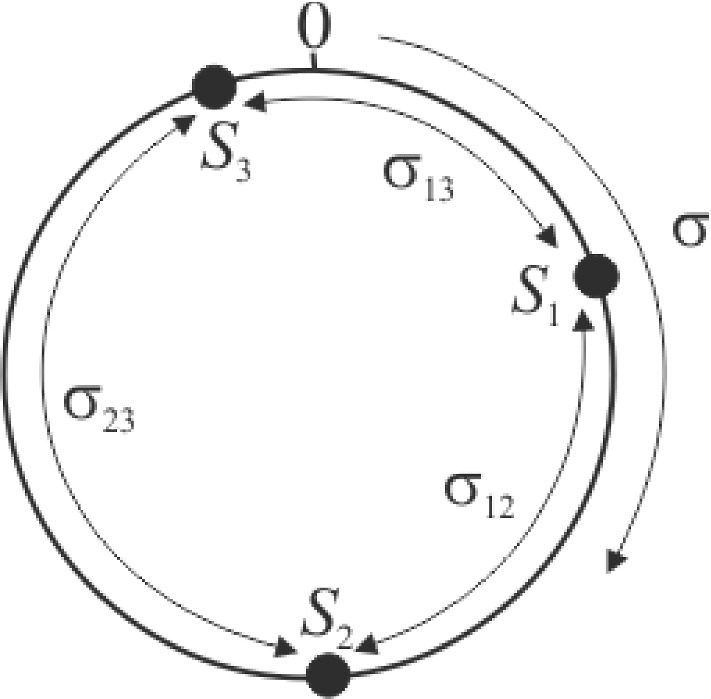
Antigenic space used in the model. The antigenic space is modelled as a circle with circumference 2. The antigenic configuration of each strain is summarised by a parameter $\sigma$ which is the location of the strain on this circle expressed as the clockwise distance from the origin. $\sigma$ takes a value between 0 and 2. The antigenic distance $\sigma _{ij}$ between two strains $S_1$ and $S_2$ is the minimum of the clockwise and anticlockwise distances between them. $\sigma _{ij}$ takes a value between 0 and 1

Antigenic configuration is parameterised by $\sigma$. This parameter is the location of the strain in a notional ‘antigenic space’ represented by a circle of circumference 2, as shown in Fig. 1. $\sigma$ is the clockwise distance around the circle from a fixed origin (Adams and Sasaki 2007; Dawes and Gog 2002). An advantage of a circular construction over a straight line is that it avoids anomalous effects propagating from the end points. The ‘antigenic distance’ between two strains is defined as the minimum of the clockwise and anticlockwise distances between them. This distance is always between 0 and 1. Cross-immunity is weaker between more distant strains.

We define the *strain community* to be the set of all strains in the combined tick, mouse and bird populations and the *infection community* to be the set of all strains in a single tick, mouse or bird. We partition the mouse, bird and nymph populations according to infection community. All larvae are free from infection with any strain because we assume there is no vertical transmission and larvae progress to the nymph stage immediately after encountering a host. For *n* strains, there are $2^n$ possible infection communities, including a null community which corresponds to the uninfected state. We encode these communities as binary strings of length *n* with a 1 in place *j* indicating that strain *j* is present. We label the population partitions $M_i, B_i, N_i$ for $i = 0 .. 2^n-1$ where partition *i* corresponds to the infection community given by the binary representation of *i*. So, for a state variable such as $M_i$, the integer label *i* can also be interpreted as the strain community set of that partition. The correspondences for $n = 4$ are shown in Table S1 of Supplementary Information.

#### Transmission Dynamics: Tick to Host

An infected nymph may transmit all or part of its infection community to a mouse or bird that it bites. We define $Q^M_{i,j,k}$ to be the probability that an encounter between a nymph with infection community *i* and a mouse with infection community *j* results in the mouse having infection community *k*. When $k = j$, the encounter does not result in transmission, so the infection community of the mouse is unchanged. Hence, for any given *i*, *j*, the sum over *k* of $Q^M_{i,j,k}$ is 1. For many community triples *i*, *j*, *k*, $Q^M_{i,j,k} = 0$ because the strains in *k* are not contained in the union of the strains in *i* and *j*. For other community triples *i*, *j*, *k*, we break down the construction of $Q^M_{i,j,k}$ into distinct components of exposure and infection.

**Fig. 2:**
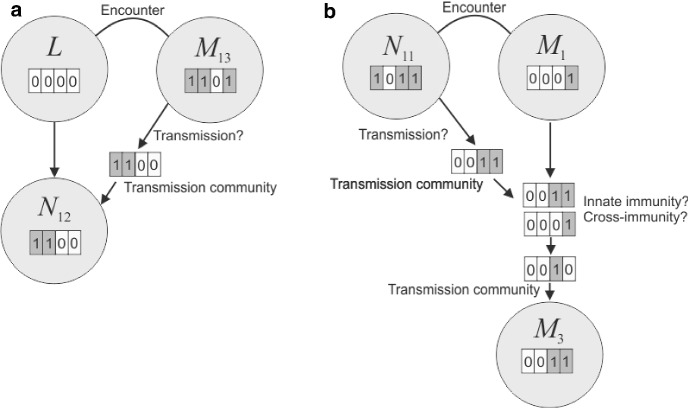
Example of the model transmission process. **a** Encounter between a larva, always uninfected, and a mouse in state $M_{13}$. The binary representation of 13 is 1101, so this mouse is infected with strains 1, 2, 4. We test for transmission and get a positive outcome. So we select the transmission community from the mouse’s infection community. In this case, it is strains 1, 2, and the larva becomes a nymph infected with these strains, so in state 1100, which corresponds to integer label 12. **b** Encounter between a nymph infected with strains 1, 3, 4 and a mouse infected with strain 4. We test for transmission and get a positive outcome. So we select the transmission community from the nymph’s infection community, in this case it is strains 3, 4. Strain 4 is already present in the mouse. So we need only consider transmission of strain 3. We test for innate immunity, summarised by the host specialisation parameter $\omega _3$, and cross-immunity between strains 3 and 4, summarised by the antigenic distance $\sigma _{34}$. We find that strain 3 is transmitted and update the mouse’s infection community to strains 3 and 4

An example of the transmission process is shown in Fig. 2. More generally, the mouse is exposed to a (non-proper) subset of the nymph’s infection community *i*, which we call the *transmission community* and label *l*. We define $A^N_{i,l}$ to be the probability that the transmission community is *l* if the infection community is *i*. Clearly, $A^N_{i,l} = 0$ if *l* is not a subset of *i*. Otherwise, we assume that if *i* is non-empty, *l* is non-empty with probability $\xi$, regardless of how many strains are in the infection community. This assumption ensures that a nymph infected with multiple strains is not more infectious than a nymph infected with one strain (Lipsitch et al. 2009). We set all non-null subsets of the infection community to be equally likely. So, if the infection community contains *m* strains, each non-null transmission community has probability $1/(2^m - 1)$. Implicitly, there is some degree of competition, or transmission bottleneck. The probability that any given strain is in the transmission community is $2^{m-1}/(2^{m}-1)$, which is 1 when $m = 1$ and decreases towards 1/2 as *m* increases. If more strains are present, each one is less likely to be transmitted. The model can also be formulated so that all strains in the infection community join the transmission community. In this case, there is no competition for transmission.

**Fig. 3:**
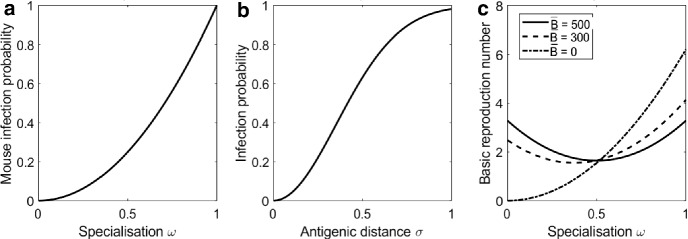
**a**, **b** Multiplicative reduction in probability of transmission to mice as a function of strain specialisation $\omega$ and antigenic distance to the nearest strain in an existing infection $\sigma$. **c** Basic reproduction number as a function of strain specialisation $\omega$ when the mouse population size $\bar{M} = 500$ and the bird population size $\bar{B} = 500, 300, 0$. Other parameters as in Table 1

The mouse is exposed to the entire transmission community. We let $C^M_{l,j,k}$ be the probability that exposure to transmission community *l* changes the mouse infection community from *j* to *k*. Each strain *s* in *l* joins *j* with independent probability $\omega _s^2(1 - e^{-2 \sigma _{sj}})$ where $\omega _s$ is the specialisation trait of strain *s* and $\sigma _{sj}$ is the minimum antigenic distance between *s* and the strains in community *j*. The term $\omega _s^2$ means that the probability of successful infection is a monotonic, concave down function of the specialisation trait (see Fig. 3a). The term $1 - e^{-2 \sigma _{sj}}$ means that the probability of successful infection is a monotonic, concave down function of the antigenic distance to the most similar strain in the existing infection community (see Fig. 3b). This shape emphasises the penalty for being antigenically close to existing strains. It is difficult to find empirical evidence to inform the shape of these function, so we have chosen generic forms. For a general discussion of how the shapes of the specialisation and cross-immunity functions influence strain interactions and coexistence see Wilson and Yoshimura (1994) and Adams and Sasaki (2007).

The probability that an encounter between a nymph with infection community *i* and a mouse with infection community *j* results in the mouse having infection community *k* is then $Q^M_{i,j,k} = \sum _{l} A^N_{i,l}C^M_{l,j,k}$ where the sum is taken over all subsets of *i*, including the empty set. A matrix of parameters $Q^B_{i,j,k}$ is defined in a similar way for transmission following encounters between nymphs and birds. The only difference is that the transmission penalty due to specialisation is $(1-\omega _s)^2$, instead of $\omega _s^2$.

#### Transmission Dynamics: Host to Tick

An infected mouse or bird may transmit all or part of its infection community to a larva that bites it. The transmission model is simpler than from nymphs to hosts because larvae always have an empty infection community. Let $Q^T_{i,0,k}$ be the probability that an encounter between a mouse or bird with infection community *i* and a larva results in a larva with infection community *k*. As before, we assume that with probability $\xi$, the larva is exposed to a non-null transmission community chosen from *i*, and each subset of *i* has the same probability, $1/(2^m - 1)$ for an infection community of *m* strains, of forming the transmission community. In contrast to before, all strains in the transmission community join the larva infection community *k* with probability 1. In the model, larvae progress to the nymph stage immediately after a host encounter.

Given $Q^M_{i,j,k}, Q^B_{i,j,k}, Q^T_{i,0,k}$, it is straightforward to write down the differential equation system (1) for the model with *n* strains and $K = 2^n$ strain communities. For reference, the model with $n=2$ strains is given in full detail in Supplementary Information.

1 $$\begin{aligned} \frac{\mathrm{d}M_0}{\mathrm{d}t}&= \mu _M\bar{M} - \mu _M M_0 - \lambda _M \left( \sum _{i=1}^{K-1} \sum _{k=0}^{K-1}N_iM_0Q^M_{i,0,k} \right) \nonumber \\ \frac{\mathrm{d}M_k}{\mathrm{d}t}&= -\mu _M M_k + \lambda _M\left( \sum _{i=1}^{K-1} \sum _{j=0}^{K-1} N_iM_jQ^M_{i,j,k} \right) - \lambda _M \left( \sum _{i=1}^{K-1}\sum _{j=0}^{K-1}N_i M_kQ^M_{i,k,j}\right) \nonumber \\ \frac{\mathrm{d}B_0}{\mathrm{d}t}&= \mu _B\bar{B} - \mu _B B_0 - \lambda _B \left( \sum _{i=1}^{K-1} \sum _{k=0}^{K-1}N_iB_0Q^B_{i,0,k} \right) \nonumber \\ \frac{\mathrm{d}B_k}{\mathrm{d}t}&= -\mu _B B_k + \lambda _B\left( \sum _{i=1}^{K-1} \sum _{j=0}^{K-1} N_iB_jQ^B_{i,j,k}\right) - \lambda _B \left( \sum _{i=1}^{K-1}\sum _{j=0}^{K-1}N_i B_kQ^B_{i,k,j}\right) \nonumber \\ \frac{\mathrm{d}L}{\mathrm{d}t}&= \mu _T \bar{T} - \mu _T L - \sum _{i=0}^{K-1} (\lambda _M M_i + \lambda _B B_i) L \nonumber \\ \frac{\mathrm{d}N_k}{\mathrm{d}t}&= -\mu _T N_k + \delta \sum _{i=0}^{K-1} (\lambda _M M_i + \lambda _B B_i)L Q^T_{i,0,k} - \sum _{i=0}^{K-1} (\lambda _M M_i + \lambda _B B_i) N_k \end{aligned}$$

where $k = 1..K-1$ for $\mathrm{d}M_k/\mathrm{d}t$ and $\mathrm{d}B_k/\mathrm{d}t$, $k = 0..K-1$ for $\mathrm{d}N_k/\mathrm{d}t$.

## Basic Reproduction Number

The definition of the basic reproduction number $R_0$ is ‘the expected number of infections arising from a typical infected individual in an otherwise naive population’ (Diekmann et al. 2010). Here, we might think of $R_0$ as a measure of a strain’s potential to invade an entirely uninfected population. The specialisation trait of a strain affects $R_0$, but the antigenic configuration trait does not affect $R_0$ because there is no history of infection in the population. It can be shown, for instance using the next-generation matrix approach (Diekmann and Heesterbeek 2000), that the basic reproduction number of a strain with specialisation trait $\omega$ is

$$\begin{aligned} R_0^2 = \frac{\xi ^2\delta L^*(\mu _M\lambda _B^2(1-\omega )^2\bar{B} + \mu _B \lambda _M^2\omega ^2 \bar{M})}{\mu _M\mu _B(\mu _T + \lambda _B \bar{B} + \lambda _M \bar{M})}. \end{aligned}$$

Here,$\bar{M}$, $\bar{B}$ and $L^* = \mu _T\bar{T}/(\mu _T + \lambda _M\bar{M} + \lambda _B\bar{B})$ are the disease-free equilibrium population sizes. The basic reproduction number does not tell us anything about the strain interactions because, by construction, the chance of co-infection is vanishingly small. However, $R_0$ can offer some useful insight into how the fundamental viability of a single strain depends on its specialisation trait and the availability of the preferred resource in the host population. Figure 3c shows how $R_0$ depends on the specialisation trait $\omega$ for different configurations of the mouse and bird equilibrium population sizes $\bar{M}$ , $\bar{B}$. In this figure, the mouse population size is always 500. The bird population size may be 500, 300 or 0. The total population size $\bar{M} + \bar{B}$ is not conserved. For a generalist ($\omega = 0.5$), $R_0$ is not affected by the bird population size because transmission is equally likely for either host species. For a mouse specialist ($0.67< \omega < 1$), reducing the bird population size (dashed and dotted lines) increases $R_0$. In this case, there is a higher probability that a tick bite will be on a mouse and, for a mouse specialist, the probability of transmission to a mouse is higher than the probability of transmission to a bird. For a bird specialist ($0< \omega < 0.33$), reducing the bird population size reduces $R_0$ because the probability of transmission from a tick to a mouse is low.

## Results

We are interested in the characteristics of stable strain communities. In terms of our model, a stable strain community is an equilibrium state that includes two or more strains with different traits that is mathematically stable. We begin with simple, constrained models with a small number of strains. We use these models to build up layers of insight into the characteristics of stable communities before concluding our analysis with more complex, less constrained models.

### Two Strains

Here, we examine how specialization, cross-immunity and the environment, in terms of the composition of the host population, determine the characteristics of stable communities of two strains. Strain 1 is always a generalist ($\omega _1 = 0.5$) at antigenic location $\sigma _1 = 0$. Strain 2 may be anything from a generalist to a strong mouse specialist ($0.5 \le \omega _2 \le 1$) at antigenic location $0 \le \sigma _2 \le 1$. The host population is structured by fixing the mouse population size at $\bar{M} = 500$ and considering bird population sizes $\bar{B}$ between 0 and 500. Note that extending strain 2 to include bird specialisation is not informative; if $\bar{B} = 500$, mouse and bird specialisation is formally equivalent; if $\bar{B} < 500$, the bird population is a depleted resource and the environment cannot support a generalist and a bird specialist.

**Fig. 4:**
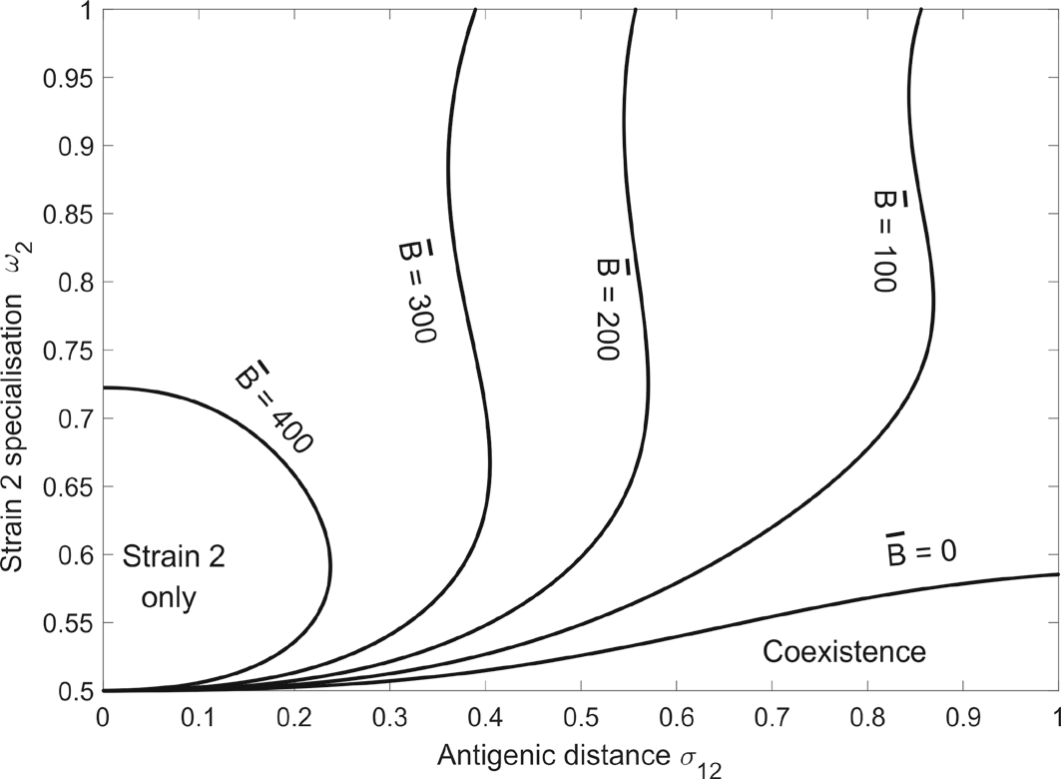
Stable equilibrium solutions for the model with $n = 2$ strains. Strain 1 is a generalist with $\omega _1 = 0.5$. Strain 2 may be a generalist or mouse specialist, $0.5 \le \omega _2 \le 1$. The antigenic distance between the strains is $\sigma _{12}$. The mouse population size is $\bar{M} = 500$. Each line corresponds to a different bird population size $\bar{B}$. Other parameters as in Table 1. The lines indicate transcritical bifurcations. To the left of each line, strain 2 excludes generalist strain 1. To the right, there is coexistence. Computed using xppaut (Ermentrout 2002)

When $\bar{B} = 500$, there is sufficient resource for any two strains to form a stable community. When the bird population is smaller, two outcomes are possible—coexistence, or exclusion of the generalist strain 1. Figure 4 shows how these outcomes depend on the size of the bird population, the degree of specialisation of strain 2 and the antigenic distance between the strains. When the bird population is almost as large as the mouse population (e.g. $\bar{B} = 400$), the two strains form a stable community unless they are antigenically similar and strain 2 is weakly specialised to mice. In this case, the weak specialist excludes the generalist because it exploits the most abundant resource (mice) more effectively and is still able to exploit the secondary resource (birds). Cross-immunity prevents most co-infection, and so the remaining resource is insufficient to sustain the generalist. A strong specialist, however, leads to coexistence because it exploits the secondary resource (birds) less effectively, and there is enough left for the generalist to persist. Weak cross-immunity leads to coexistence because co-infection allows the same resource to be exploited by both strains.

When the bird population is substantially smaller than the mouse population (e.g. $\bar{B} = 300$), stable communities require weak cross-immunity. When co-infection is difficult, the reduced size of the secondary resource (birds) means that the generalist must also exploit the primary resource (mice) to persist, but the specialist exploits this resource more effectively. When there are no birds ($\bar{B} = 0$), stable communities are characterised by very weak specialisation because both strains have to share the primary resource. Note that even when there is no cross-immunity ($\sigma = 1$), generalists and strong specialists cannot coexist. In this case, the specialist exploits the only resource (mice) more effectively, and although co-infection allows the generalist to exploit the same resource, conservation of infection intensity maintains a competitive effect because each strain in a co-infection is less likely to be transmitted than it would be in a single infection.

### Three Strains

Here, we examine how specialization and cross-immunity determine the characteristics of stable communities of three strains.

#### Structured Strain Communities

We begin by considering a framework for the strain community structure that is sufficiently tractable to provide useful insight. Strain 1 is a generalist ($\omega _1 = 0.5$). Strain 2 may be anything from a generalist to a strong mouse specialist ($0.5 \le \omega _2 \le 1$). Strain 3 may have any specialisation ($0 \le \omega _3 \le 1$). We will say that strains are ‘aligned’ when they are specialised to the same host type. The three strains are evenly distributed over an antigenic interval of length $\psi$, with $\sigma _1 = 0$, $\sigma _2 = \psi /2$, $\sigma _3 = \psi$. So the minimum distances between strains are $\sigma _{12} = \sigma _{23} = \psi /2$ and $\sigma _{13} = \text{ min }\{\psi , 2 - \psi \}$, and $\psi$ controls the dispersion in antigenic space. Larger values correspond to less cross-immunity, and $\psi = 1.33$ sets the distance between each strain at $\sigma = 0.67$, the largest possible for three strains in our framework. We also consider switching off the antigenic interaction altogether, so there is no cross-immunity between strains. For simplicity, we keep a uniform environment with equal sized mouse and bird populations ($\bar{M} = \bar{B} = 500$).

We solve system (1) with three strains taking trait values across the stated ranges. For the initial condition, we take the disease-free equilibrium and switch 5 mice and 5 birds from the uninfected state into each of states $M_7$ and $B_7$, i.e. infected with all strains. In addition, we switch a random number, uniformly distributed between 0 and 1, of mice and birds from the uninfected state into each infection state $M_i$, $B_i$ for $i = 1..7$. We solve the system in time blocks of 1000 years. We stop the computation when the largest difference in any state variable between $t = 0$ and $t=1000$ in a block is less than 0.15. We assume the system has then reached a stable equilibrium. At this point, we consider a strain to be present if the total number of mice and birds with an infection community which includes that strain is greater than 1. If all three strains are still present, then we consider the original community stable. Otherwise, we consider it unstable.

**Fig. 5:**
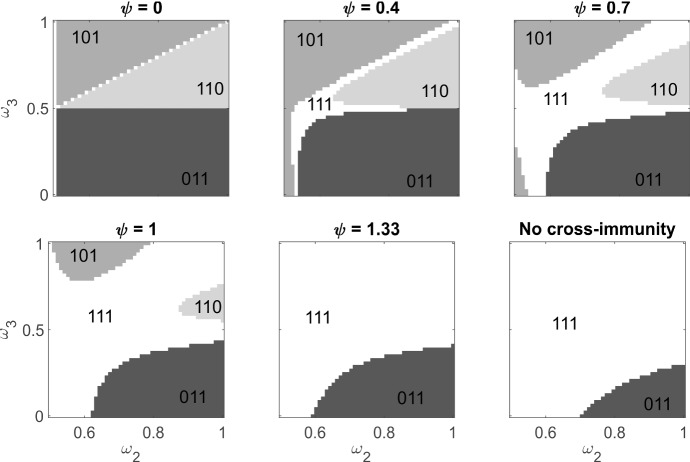
Stable equilibria for the model with $n = 3$ strains. Strain 1 is a generalist with $\omega _1 = 0.5$. Strain 2 may be a generalist or mouse specialist, $0.5 \le \omega _2 \le 1$. Strain 3 may be a generalist, mouse or bird specialist $0 \le \omega _3 \le 1$. The three strains are evenly distributed over an antigenic interval of length $\psi$ such that the minimum distances between strains are $\sigma _{12} = \sigma _{23} = \psi /2$ and $\sigma _{13} = \text{ min }\{\psi , 2 - \psi \}$. All possible transmission communities have equal probability. Shades correspond to different equilibrium outcomes, as labelled by the binary community representation, e.g. 110 indicates strains 1 and 2 coexist, and strain 3 is excluded. Mouse and bird populations sizes are both 500. Other parameters as in Table 1. Computed by numerical solution of system (1) using MATLAB

Figure 5 shows how community stability depends on strain specialisation and antigenic dispersion. When cross-immunity prevents any co-infection ($\psi = 0$), there is no stable community of three strains. A pairing of any (mouse specialised) strain 2 and any bird specialised strain 3 ($\omega _3 < 0.5$) excludes the generalist strain 1. Here, the specialists exert strong competition on the generalist for each resource. A pairing in which both strains 2 and 3 are specialised to mice results in the exclusion of the less specialised of these two strains. In this case, the generalist experiences only weak competition for the bird resource, but there is strong competition between all three strains for the mouse resource.

Antigenic dispersion facilitates stable communities of three strains because co-infection allows multiple use of the same resources. Weak differentiation ($\psi = 0.4$) admits stable communities composed of three generalist strains ($\omega$ close to 0.5). Increasing antigenic differentiation cracks open this region of coexistence, admitting more specialised strains into these communities and facilitating stable communities composed of two generalists and a specialist or a generalist and two aligned specialists. When there is substantial antigenic variation ($\psi = 1.33$), or no cross-immunity at all, the only communities that are unstable are those composed of a generalist, strong mouse specialist and strong bird specialist. The generalist is excluded. The implicit competition for transmission is an important factor in the prevention of coexistence. If this competition is removed, so the entire infection community is always transmitted, then widespread coexistence occurs when the antigenic dispersion is much lower. Figure S1 shows how community stability depends on strain specialisation and antigenic dispersion when there is no implicit competition.

#### Unstructured Strain Communities

We now consider communities of three strains in which traits are randomly assigned without any constraints. Our objective is to characterise stable communities in terms of the distribution of strains in the trait spaces of specialisation and antigenic configuration.

We generated communities of three strains with specialisation traits $\omega _i$ and antigenic configuration traits $\sigma _i$, for $i = 1, 2, 3$, chosen uniformly at random from [0, 1] and [0, 2], respectively. We initially characterise these communities by the specialisation weight $\hat{\omega }$ and the antigenic dispersion $\hat{\sigma }$. We define $\hat{\omega }$ to be the root-mean-square distance of the $\omega$ traits from 0.5, $\hat{\omega } = \sqrt{\sum _i (\omega _i - 0.5)^2}/3$. This statistic provides a coarse measure of the degree of specialisation in the community. A value of 0 indicates that all strains are pure generalists, and 0.5 indicates that all strains are pure specialists. We define $\hat{\sigma }$ to be the circular variance of the $\sigma$ traits. For each strain, we define $\theta _i = -\sigma _i\pi$ if $\sigma _i < 1$ and $(2-\sigma _i)\pi$ if $\sigma _i > 1$, $\bar{c} = \sum _i \cos (\theta _i)$, $\bar{s} = \sum _i \sin (\theta _i)$. Then, $\hat{\sigma } = 2(1 - \sqrt{\bar{c}^2 + \bar{s}^2})$. This statistic is a measure of the variance in a set of values when there is no definitive orientation. $\hat{\sigma }= 0$ indicates that all values are identical, and $\hat{\sigma } = 2$ indicates that the values are equally spaced with the maximum possible distance between them.

**Fig. 6:**
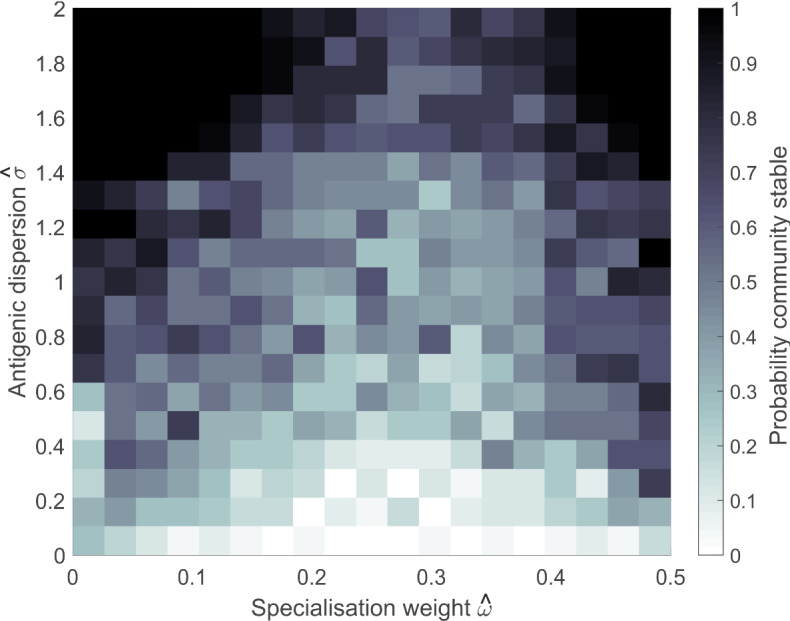
Probability that randomly generated three strain communities are stable, depending on specialisation weight and antigenic dispersion. For each grid square, 25 communities were generated with trait values consistent with the given specialisation weight $\hat{\omega }$ and antigenic dispersion $\hat{\sigma }$. For each community, system (1) was solved to equilibrium and the community considered stable if all three strains were present at equilibrium

In order to assess the relationship between specialisation weight, antigenic dispersion and community stability, we constructed a $20\times 20$ grid of $\hat{\sigma }$ and $\hat{\omega }$ values. We generated communities of three strains with random uniform trait values, calculated their $\hat{\sigma }$ and $\hat{\omega }$ values and assigned them to the appropriate grid box. We continued generating communities until each grid box contained exactly 25 communities. For each community, we solved system (1) to equilibrium as described above. A community was considered stable if all three strains were present at equilibrium. Figure 6 shows the proportion of communities in each grid box that were stable. When antigenic dispersion is low, stable communities are rare. As dispersion increases, we begin to find stable communities strongly weighted to generalism or to specialism. When dispersion is high, stable communities are common, but a substantial proportion of communities with intermediate specialisation weights are still unstable. These are communities composed of a generalist and two specialists, or three intermediate specialists. When there is no implicit competition for transmission, the general patterns are similar, but the signal is much weaker. See Figure S2 in Supplementary Information.

In order to assess how the well specialisation weight and antigenic dispersion predict community stability, we constructed a decision tree, using the R implementation of C5.0 (Quinlan 1993) with 10 trial boosting, to classify communities as stable or unstable according to the values of $\hat{\sigma }$ and $\hat{\omega }$. However, this algorithm was unable to find a partition on $\hat{\omega }$ that effectively classified stability for intermediate antigenic dispersion. This uncertainty is consistent with the results shown in Fig. 6. In search of an improved classifier, we considered an alternative community characterisation based on the number of specialists $N_S$, the specialisation alignment $A_S$ and the antigenic dispersion $\hat{\sigma }$. We chose a specialisation threshold $\omega _s$ and let $N_M$ to be the number of mouse specialists, strains with $\omega _i$ such that $1-\omega _s< \omega _i < 1$, and $N_B$ to be the number of bird specialists, strains with $\omega _i$ such that $0< \omega _i<\omega _s$. Then, $N_S =N_M + N_B$ is the total number of specialists and the specialisation alignment $A_S = \text{ max }\{N_M, N_B \}/{N_S}$ indicates the extent to which these strains are aligned, i.e. specialised to the same host resource. Numerical experiments determined that a specialisation threshold of $\omega _s = 0.33$ performed well.

**Fig. 7:**
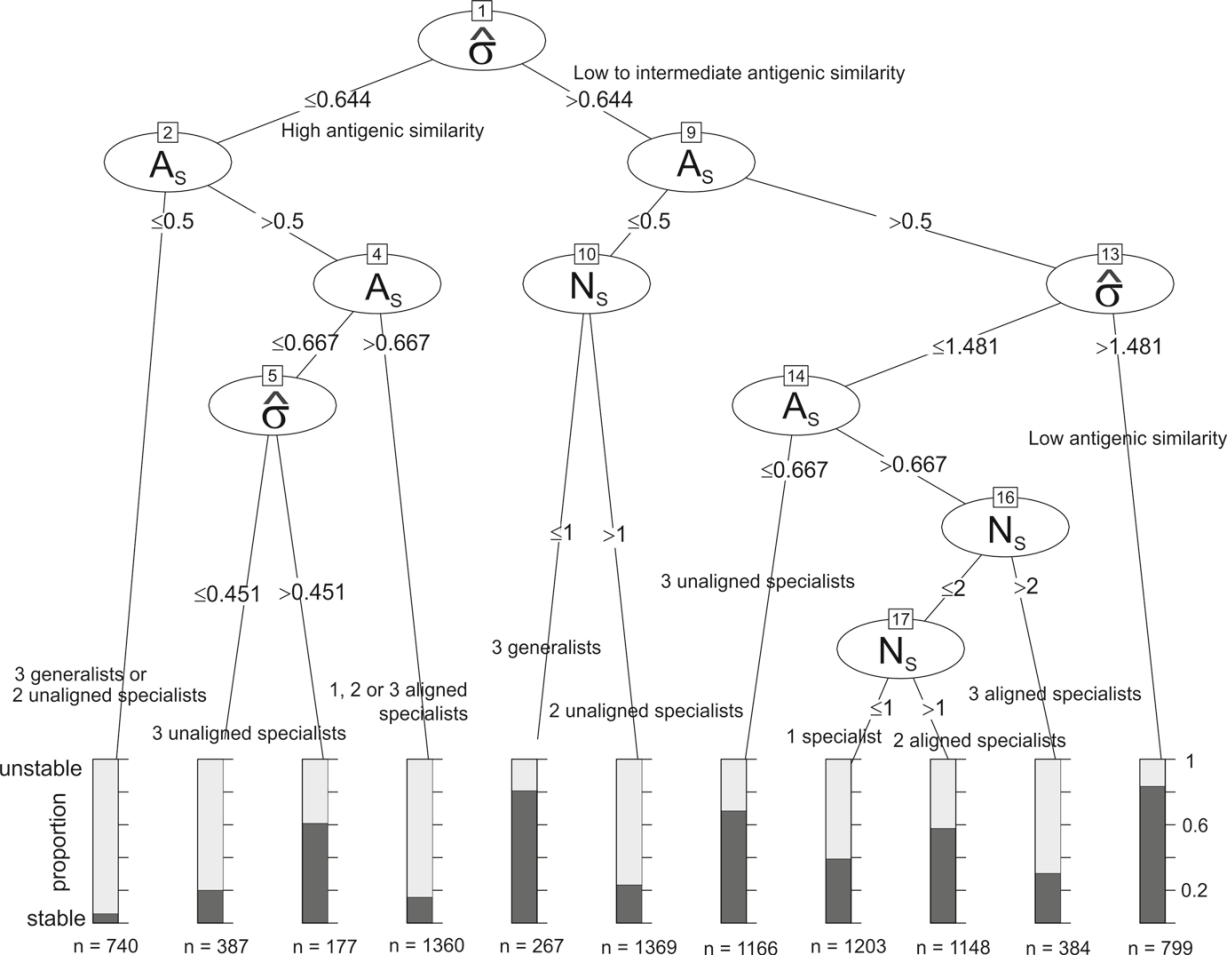
Decision tree classifying stability of three strain communities based on the number of specialists $N_S$, the specialisation alignment $A_S$ and the antigenic dispersion $\hat{\sigma }$. Generated using C5.0 with a training set of 9000 out of $10^5$ random trait combinations of which 3977 produced stable three strain communities

We constructed a decision tree, again using C5.0 with 10 trial boosting, to classify communities as stable or unstable according to the number of specialists, the balance of specialists and the circular variance of the antigenic configuration. The classifier was 75 % accurate. The decision tree is shown in Fig. 7. The algorithm partitions the communities into 11 groups as follows:

- Low antigenic dispersion, 3 generalists or 2 unaligned specialists. Unstable (6.1% error).
- Very low antigenic dispersion, 3 unaligned specialists. Unstable (20.6 % error).
- Low antigenic dispersion, 3 unaligned specialists. Stable (38.5 % error).
- Low antigenic dispersion, 1, 2 or 3 aligned specialists. Unstable (15.5 % error).
- Intermediate-high antigenic dispersion, 3 generalists. Stable (18.9 % error).
- Intermediate-high antigenic dispersion, 1 generalist and 2 unaligned specialist. Unstable (22.9% error).
- High antigenic dispersion, 3 unaligned specialists. Stable (31.9% error).
- High antigenic dispersion, 2 generalists and 1 specialist. Unstable (39.6% error)
- High antigenic dispersion, 1 generalists and 2 aligned specialists. Stable (42.5% error)
- High antigenic dispersion, 3 aligned specialists. Unstable (28.7% error).
- High antigenic dispersion, except 3 generalists or 1 generalists and 2 unaligned specialists. Stable (17.1 % error)

Only a small minority of communities with $\hat{\sigma } < 0.64$ were stable, and most of these were composed of 3 unaligned specialists. When $\hat{\sigma } > 0.64$, the majority of communities composed of 3 generalists were stable, and the majority of communities composed of a generalist and 2 unaligned specialists were unstable. Of the remaining communities, most were stable when $\hat{\sigma } > 1.48$. However, for intermediate dispersion ($0.64< \hat{\sigma } < 1.48$) the algorithm was not able to efficiently classify the communities based on our summary characteristics. These results are in broad agreement with the insights from the structured approach summarised in Fig. 5.

#### Four or More Strains

**Fig. 8:**
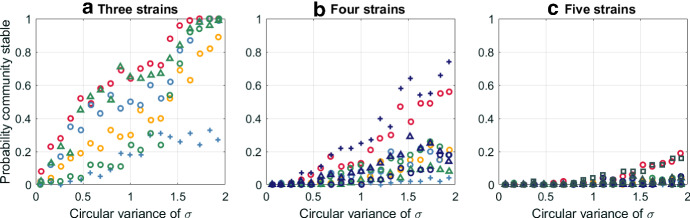
Probability that communities of three, four and five strains are stable depending on the number of specialists, specialisation alignment and antigenic dispersion. Each point corresponds to 100 communities with randomly generated trait values consistent with the given characterisation. System (1) was solved to equilibrium with each of these trait combinations. A community was considered stable if all strains had nonzero prevalence at equilibrium. Marker type and colour indicate the number of specialists and their alignment. Red—0. Yellow—1. Light blue—2 (o aligned, + unaligned 1:1). Green—3 (o aligned, $\triangle$ unaligned 2:1). Dark blue—4 (o aligned, + unaligned 2:2, $\triangle$—unaligned 3:1). Dark grey—5 (o aligned, $\triangle$ unaligned 4:1, $\square$ unaligned 3:2) (Color figure Online).

In order to examine how our characterisation statistics relate to the stability of larger communities, we considered communities of four, five and six strains with randomly generated traits. We placed each community into one of 20 groups according to the antigenic dispersion value, and then a subgroup according to the number of specialists and specialisation alignment. We generated a total of 100 communities in each subgroup. As before, for each community we solved system (1) to equilibrium and the community was considered stable if all strains persisted. The probability that a community in each subgroup was stable is shown in Fig. 8.

For three strains, communities of 3 generalists or 3 unaligned specialists had the highest probability of being stable; communities of 1 generalist and 2 unaligned specialists or 3 aligned specialists had the lowest probability of being stable. Increasing the antigenic dispersion increases the stability probability for all community types. But even with maximum dispersion, the majority of communities with 1 generalist and 2 unaligned specialists are unstable. With four strains, the antigenic niche space is becoming saturated and the majority of communities were unstable even with maximum antigenic dispersion. Communities of 4 generalists or 4 specialists aligned equally between each host type have the highest probability of being stable. For five strains, stable communities are rare and almost entirely limited to 5 generalist or 5 specialists aligned as equally as possible between the two host types. For six strains, it is almost impossible to find stable communities by random trait generation, although they can be constructed with an evolutionary algorithm that generates new strains to exploit vacant niche spaces. This method generally produces stable six strain communities composed of 3 specialists aligned to each host type, with maximum antigenic dispersion within each of the specialist groups. We will explore the evolutionary dynamics in detail in future work.

## Discussion

We have used a mathematical model that combines ecological and epidemiological processes with strain interactions to gain insight into how the combination of host specialisation and immune cross-reaction shapes microbial strain diversity when transmission is mediated by a generalist vector. We based our work on *Borrelia burgdorferi*, circulating in mice and birds, transmitted by tick larvae and nymphs, but the framework is quite general.

We have shown that if there is little antigenic differentiation between strains, stable parasite communities are usually composed entirely of generalists or of specialists roughly balanced between the host types. Antigenic differentiation between strains introduces an additional niche dimension that cracks open the host specialisation niches. Increasing antigenic differentiation facilitates a greater diversity of stable communities. Straightforward and definitive rules to characterise the trait compositions of stable communities are elusive. But, in broad terms, they are usually composed entirely of generalists, or generalists with similar specialists aligned to the same host type, or specialists balanced between host types. When antigenic differentiation is high, stable communities exist across almost the whole range of specialisation traits. However, communities with generalists and strong specialists aligned to different host types are rare; generalists are usually excluded. Of course, all of our results have focussed on small strain communities. In reality, 15 or more Bb ospC types may co-circulate. As it stands, stable communities as large as this do not occur in our model. The notional circular antigenic space and mapping between antigenic distance and cross-protective immunity that we used constrain the number of antigenic niches. The true space in which Bb antigenic variation occurs, and the relationship between antigenic composition and immunological interactions, is poorly understood. However it is likely to be high dimensional and complex, leading to more intricate antigenic niche structures and larger stable communities. We will use agent-based simulation to explore some of these possibilities in future work but anticipate that the fundamental understanding of the determinants of community stability we have developed in this paper will continue to apply.

In formulating our model, we kept the ecology fairly simple in order to keep the analysis reasonably tractable. In future work, we will examine how the broad rules of community composition emerging from this model are affected by several factors known to be important in the eco-epidemiology of Borrelia. Our model assumes the only transmission pathway is host–tick–host. However, co-feeding transmission allows Borrelia strains to be transferred directly between ticks feeding on the same host. This process may increase the prevalence of high strain multiplicity co-infections within nymphs. Our model assumes that all hosts contact ticks at the same rate. However, observed distributions of ticks over host populations in the field are consistent with aggregation. Some hosts carry disproportionately high numbers of ticks. Heavily burdened individual hosts may increase the prevalence of high strain multiplicity co-infections. Our model assumes that there is no seasonal variation in population dynamics or behaviour, and ticks progress to the next life-history stage immediately after a host encounter. However, Borrelia eco-epidemiology has complex seasonal drivers. Vertebrate host populations may have spring and summer reproductive pulses and winter dormancy. The development rates and questing behaviour of ticks depend on temperature, and they typically enter a diapaused state in winter. The interplay of seasonal drivers is such that, in different regions, the tick life cycle may take one year, two or even three years, and several distinct phase relationships between tick life stages and host population trajectories are been observed (Kurtenbach et al. 2006). These factors may affect how co-infections are accumulated and maintained.

We have considered the ecological stability of communities formed by one-off random assembly; evolutionary processes do not feature on our model. We expect communities assembled by sequences of invasion and replacement events, resulting from diverse strains circulating geographically or novel strains being repeatedly generated by mutation, to exploit the niche space more efficiently. Emergent community structures may, however, be disrupted by recombination. Haven et al. (2011) argue that all Bb ospC groups in the north-eastern USA constitute a single generalist ecotype and sympatric divergence to host specialisation is unlikely. Their argument is based on genomic analysis and a codon-based simulation of genome evolution. This model shows that high recombination rates prevent adaptive evolution of host specialisation, but negative frequency-dependent selection can still maintain allelic diversity. Our model, at this stage, does not offer any insight into evolutionary stability. But there is mounting empirical evidence of host specialisation among Bb strains (Lin et al. 2020), and the puzzle of how this diversity is generated and maintained remains to be resolved. Our framework provides the ecological context and understanding in which to explore these evolutionary dynamics.

## Conclusion

In conclusion, we have found that the interaction of cross-immunity and host specialisation traits creates a intricate niche structure. Definitive rules that describe this niche structure and map it to the trait characteristics of stable strain communities are hard to pin down. However, we have identified broad patterns that summarise how the specialisation traits of stable communities change under increasing antigenic differentiation. The geographic distribution of *B. burgdorferi* has been shaped by climate change, habitat fragmentation and species loss. Identifying the ecological mechanisms maintaining *B. burgdorferi* diversity may also provide insight into how historic and future human-mediated environmental change shapes not only the geographic range but also the diversity of this important zoonotic pathogen.

## Supplementary Information

Below is the link to the electronic supplementary material.

Supplementary material 1 (pdf 454 KB)

## Data Availability

All MATLAB and R code used in this study are available on request from BA.
