# Supplementary material for: Host Specialisation, Immune Cross-Reaction and the Composition of Communities of Co-circulating Borrelia Strains
Source: Bull Math Biol. 2021 May 3;83(6):66. doi: 10.1007/s11538-021-00896-2 (PMC8093179; doi:10.1007/s11538-021-00896-2)
Supplement: Supplementary file 1 — Supplementary material 1 (pdf 454 KB) [file 11538_2021_896_MOESM1_ESM.pdf]

Supplementary Information
Host specialisation, immune cross-reaction and the composition of communities of co-circulating Borrelia strains

Ben Adams, Katharine S Walter, Maria A Diuk-Wasser

Two strain model

In the model with two strains, the possible infection communities are 0 (00, uninfected), 1 (01, infected with strain 2 only), 2 (10, infected with strain 1 only), 3 (11, infected with strains 1 and 2). The equations for the mice, larvae and nymphs are as follows. The equations for the birds are similar.

$$
\begin{aligned}
\frac{dM_0}{dt} &= \mu_M \bar{M} - \mu_M M_0 - \lambda_M M_0 \left(Q^M_{1,0,1} N_1 + Q^M_{2,0,2} N_2 + \sum_{k=1}^{3} Q^M_{3,0,k} N_3 \right) \\
\frac{dM_1}{dt} &= -\mu_M M_1 + \lambda_M M_0 \left(Q^M_{1,0,1} N_1 + Q^M_{3,0,1} N_3 \right) - \lambda_M M_1 \left(Q^M_{2,1,3} N_2 + Q^M_{3,1,3} N_3 \right) \\
\frac{dM_2}{dt} &= -\mu_M M_2 + \lambda_M M_0 \left(Q^M_{2,0,2} N_2 + Q^M_{3,0,2} N_3 \right) - \lambda_M M_2 (Q^M_{1,2,3} N_1 + Q^M_{3,2,3} N_3) \\
\frac{dM_3}{dt} &= -\mu_M M_3 + \lambda_M \left[M_0 Q^M_{3,0,3} N_3 + M_1 \left(Q^M_{2,1,3} N_2 + Q^M_{3,1,3} N_3 \right) + M_2 \left(Q^M_{1,2,3} N_1 + Q^M_{3,2,3} N_3 \right) \right] \\
\frac{dL}{dt} &= \mu_T \bar{T} - \mu_T L - \left(\lambda_M \sum_{i=0}^{3} M_i + \lambda_B \sum_{i=0}^{3} B_i \right) L \\
\frac{dN_0}{dt} &= -\mu_T N_0 + \delta \left[\lambda_M M_0 + \lambda_B B_0 + (1-\xi) \left(\lambda_M \sum_{i=1}^{3} M_i + \lambda_B \sum_{i=1}^{3} B_i \right) \right] L - \left(\lambda_M \sum_{i=0}^{3} M_i + \lambda_B \sum_{i=0}^{3} B_i \right) N_0 \\
\frac{dN_1}{dt} &= -\mu_T N_1 + \delta \left[(\lambda_M M_1 + \lambda_B B_1) Q^T_{1,0,1} + (\lambda_M M_3 + \lambda_B B_3) Q^T_{3,0,1} \right] L - \left(\lambda_M \sum_{i=0}^{3} M_i + \lambda_B \sum_{i=0}^{3} B_i \right) N_1 \\
\frac{dN_2}{dt} &= -\mu_T N_2 + \delta \left[(\lambda_M M_2 + \lambda_B B_2) Q^T_{2,0,2} + (\lambda_M M_3 + \lambda_B B_3) Q^T_{3,0,2} \right] L - \left(\lambda_M \sum_{i=0}^{3} M_i + \lambda_B \sum_{i=0}^{3} B_i \right) N_2 \\
\frac{dN_3}{dt} &= -\mu_T N_3 + \delta \left[(\lambda_M M_3 + \lambda_B B_3) Q^T_{3,0,3} \right] L - \left(\lambda_M \sum_{i=0}^{3} M_i + \lambda_B \sum_{i=0}^{3} B_i \right) N_3
\end{aligned}
$$

where Q^M has entries of 0 except

$$\begin{aligned}
Q^M_{1,0,1} &= \xi\omega_2^2 \\
Q^M_{2,0,2} &= \xi\omega_1^2 \\
Q^M_{3,0,1} &= \xi\left(\frac{\omega_2^2 + \omega_2^2(1-\omega_1^2)}{3}\right) \\
Q^M_{3,0,2} &= \xi\left(\frac{\omega_1^2 + (1-\omega_2^2)\omega_1^2}{3}\right) \\
Q^M_{3,0,3} &= \xi\frac{\omega_1^2\omega_2^2}{3} \\
Q^M_{2,1,3} &= \xi\omega_1^2(1-\exp(-2\sigma_{12})) \\
Q^M_{3,1,3} &= \xi\left(\frac{(1-\omega_2^2)\omega_1^2 + \omega_1^2}{3}\right)(1-\exp(-2\sigma_{12})) \\
Q^M_{1,2,3} &= \xi\omega_2^2(1-\exp(-2\sigma_{12})) \\
Q^M_{3,2,3} &= \xi\left(\frac{\omega_1^2 + \omega_2^2(1-\omega_1^2)}{3}\right)(1-\exp(-2\sigma_{12}))
\end{aligned}$$

Table S1: Infection communities with corresponding binary and integer labels for $n = 4$ strains.

Binary label	Integer label	Interpretation
0000	0	Uninfected
0001	1	Strain 4 only
0010	2	Strain 3 only
0011	3	Strains 3 and 4
0100	4	Strain 2 only
0101	5	Strains 2 and 4
0110	6	Strains 2 and 3
0111	7	Strains 2, 3 and 4
1000	8	Strain 1 only
1001	9	Strains 1 and 4
1010	10	Strains 1 and 3
1011	11	Strains 1, 3 and 4
1100	12	Strains 1 and 2
1101	13	Strains 1, 2 and 4
1110	14	Strains 1, 2 and 3
1111	15	Strains 1, 2, 3 and 4

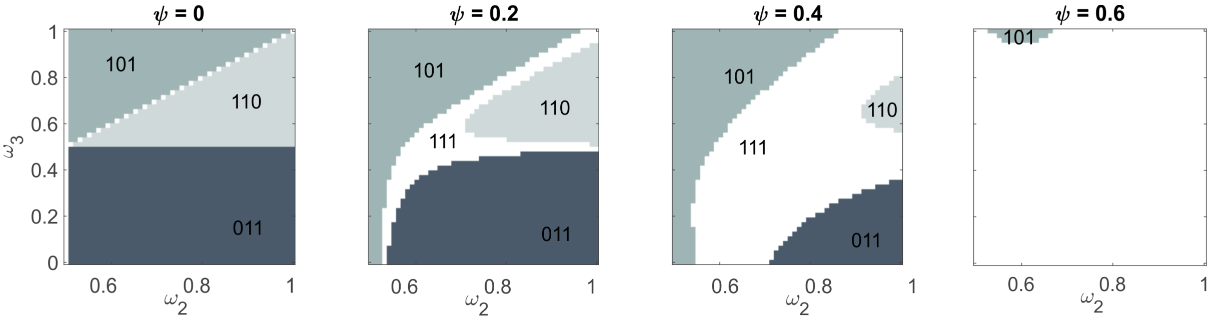

Figure S1: Stable equilibria for the model with $n = 3$ strains when the transmission community is always composed of all strains in the infection community. Strain 1 is a generalist with $\omega_1 = 0.5$. Strain 2 may be a generalist or mouse specialist, $0.5 \leq \omega_2 \leq 1$. Strain 3 may be a generalist, mouse or bird specialist $0 \leq \omega_3 \leq 1$. The three strains are evenly distributed over an antigenic interval of length ψ such that the minimum distances between strains are $\sigma_{12} = \sigma_{23} = \psi/2$ and $\sigma_{13} = \min\{\psi, 2 - \psi\}$. Shades correspond to different equilibrium outcomes, as labelled by the binary community representation e.g. 110 indicates strains 1 and 2 coexist, strain 3 is excluded. Mouse and bird populations sizes are both 500. Other parameters as in Table 1. Computed by numerical solution of system (1) using Matlab.

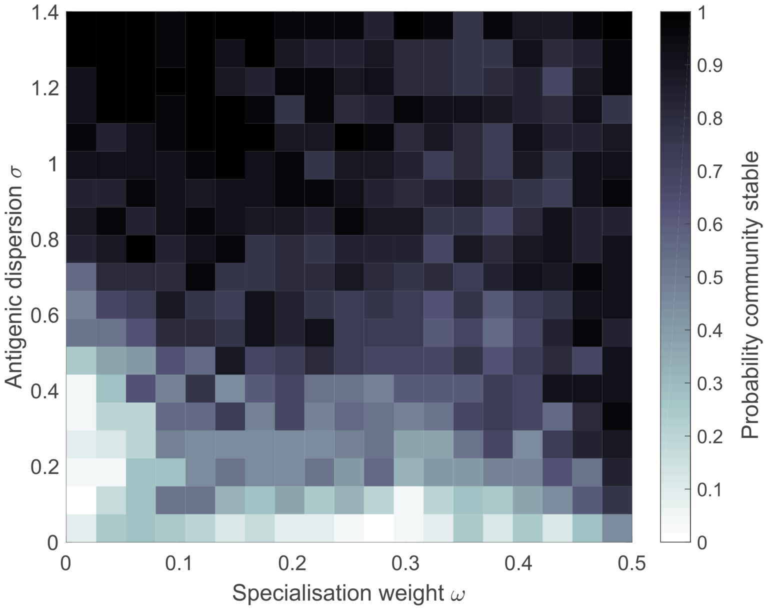

Figure S2: Probability that randomly generated three strain communities are stable, depending on specialisation weight and antigenic dispersion, when the transmission community is always composed of all strains in the infection community. For each grid square 25 communities were generated with trait values consistent with the given specialisation weight $\hat{\omega}$ and antigenic dispersion $\hat{\sigma}$. For each community, system (1) was solved to equilibrium and the community considered stable if all three strains were present at equilibrium.

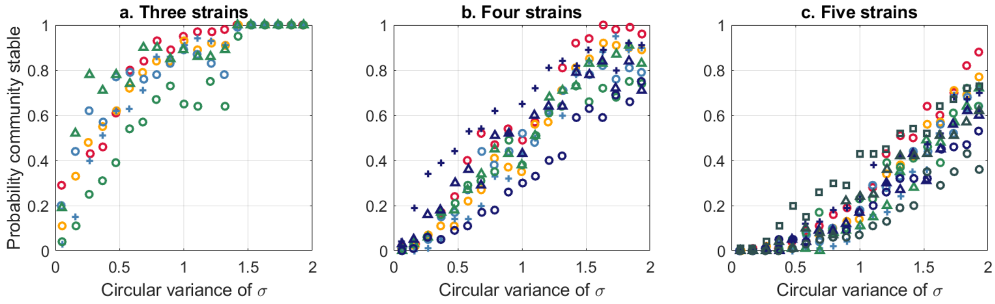

Figure S3: Probability that communities of three, four and five strains are stable depending on the number of specialists, specialisation alignment and antigenic dispersion when the transmission community is always composed of all strains in the infection community. Each point corresponds to 100 communities with randomly generated trait values consistent with the given characterisation. System (1) was solved to equilibrium with each of these trait combinations. A community was considered stable if all strains had non-zero prevalence at equilibrium. Marker type and colour indicate the number of specialists and their alignment. Red - 0. Yellow - 1. Light blue - 2 (o aligned, + unaligned 1:1). Green - 3 (o aligned, △ unaligned 2:1). Dark blue - 4 (o aligned, + unaligned 2:2, △ - unaligned 3:1). Dark grey - 5 (o aligned, △ unaligned 4:1, □ unaligned 3:2).
